# Dental age assessment in the living: a comparison of two common stage classifications for assessing radiographic visibility of the root canals in mandibular third molars

**DOI:** 10.1007/s00414-023-03121-y

**Published:** 2023-11-11

**Authors:** Maximilian Timme, Jan Viktorov, Laurin Steffens, Adam Streeter, André Karch, Andreas Schmeling

**Affiliations:** 1 Institute of Legal Medicine, University Hospital Münster, Röntgenstraße 23, 48149 Münster, Germany; 2 Institute of Epidemiology and Social Medicine, University of Münster, Domagkstraße 3, 48149 Münster, Germany

**Keywords:** Age assessment, Dental age, Degenerative characteristics, Third molar, Orthopantomogram

## Abstract

After dentition is complete, degenerative tooth characteristics can be used for dental age assessment. Radiological assessment of the visibility of the root canals of the mandibular third molars in dental panoramic radiographs (DPRs) is known to be one such suitable feature. Essentially, two different stage classifications are available for evaluating the visibility of the root canals of mandibular third molars in the DPR. The aim of this study was to determine if one method outperforms the other. Therefore, the 2010 method of Olze et al. was directly compared to the 2017 method of Lucas et al. in the 2020 modification of Al Qattan et al. To this end, 233 DPRs from 116 females and 117 males aged 20.0 to 40.9 years were evaluated by three independent experienced examiners. In addition, one examiner ran two independent evaluations. Correlation between age and stage was investigated, and the inter- and intra-rater reliability was estimated for both methods. Correlation between age and stage was higher with the Olze method (Spearman rho 0.388 [95% CI 0.309, 0.462], males and 0.283 [95% CI 0.216, 0.357], females) than the Lucas method (0.212 [95% CI 0.141, 0.284], males and 0.265 [95% CI 0.193, 0.340], females). The intra-rater repeatability of the Olze method (Krippendorff’s *α* = 0.576 [95% CI 0.508, 0.644], males and *α* = 0.592 [95% CI 0.523, 0.661], females) was greater than that for the Lucas method (intra-rater *α* = 0.422 [95% CI 0.382, 0.502], males and *α* = 0.516 [95% CI 0.523, 0.661], females). Inter-rater reproducibility was also greater for the Olze method (*α* = 0.542 [95% CI 0.463, 0.620], males and *α* = 0.533 [95% CI 0.451, 0.615], females) compared to the Lucas method (*α* = 0.374 [95% CI 0.304, 0.443], males and *α* = 0.432 [95% CI 0.359, 0.505], females). The method of Olze et al. was found to present marginal advantages to the Lucas et al. method across all examinations and may be a more appropriate method for application in future studies.

## Introduction

Forensic age assessment of living persons can, with a well-known, statistical margin of uncertainty, determine the chronological age of a person [1, 2]. By doing so, forensic age assessment today is able to regularly show that legally relevant age limits have been passed with a probability bordering on certainty [1]. Thus, forensic age assessment may represent an effective means of ensuring age-appropriate proceedings. This includes, in particular, securing the special status of unaccompanied minor refugees [3–6].

Forensic age estimation will continue to play an important role, particularly in view of increasing migration movements and the associated rise in the number of persons of undetermined age [7]. This is also reflected in the fact that forensic age assessment has become a focus of research in the forensic sciences [8–19].

According to the Study Group on Forensic Age Diagnostics recommendations for performing age assessments, an examination of the dental status should always be included [20]. Dental panoramic radiographs (DPRs; also PAN or orthopantomograms (OPGs) [21]) are routinely used for this purpose. The mineralization and eruption of the third molars are assessed, and the findings are compared with a reference population [22, 23]. However, when the legal age of majority is determined by a limit at 18 years [24], this poses a challenge with the use of tooth development for evaluating age, since tooth development, including the third molar, can be completed before the age of 18 [25]. Thus, the detection of this often decisive age limit with features of tooth development is not possible with the highest level of certainty. After completion of tooth development, degenerative tooth characteristics can be used for age assessment [26–29]. A major disadvantage in using degenerative tooth characteristics in age assessment compared to tooth development characteristics is that they are far more susceptible to external influences. While tooth development is thought to be essentially genetically determined [30–33], degenerative tooth characteristics can be significantly influenced by diet, habit, medication, or disease [34–38]. Accordingly, it must be carefully examined in each individual case whether it is an age-associated degenerative process or a pathological process. Corresponding pathological teeth must then not be used for age assessment.

A variety of degenerative tooth characteristics have been reported in the past, which can be used for age assessment [26, 39–44]. One such feature is the decrease in visibility of the root canals or root pulp of mandibular third molars on DPR. The principal suitability of the feature for age assessment can be regarded as assured today, as evidenced by large studies [40, 45–50]. The obstruction of the root canals is a purely optical phenomenon [40]. One may suppose that the canals will actually not close completely, as this would not be compatible with the vitality of the tooth. The physiological mechanism of this phenomenon is not conclusively clarified. It can be assumed that secondary dentin formation in the pulp and the associated actual reduction in the size of the root pulp play a role [51]. On the other hand, changes in the bone or tooth structure may have an influence on the feature. For example, the decrease in diameter of dentinal tubules particularly near the pulp and the accompanying densification of dentin over the course of life have been documented [44, 52, 53]. This may influence the radiopacity of the dentin and thus the radiological representation of the fine root pulp. Increases in the amount of fiber in the pulp can also have a radiographic effect [53], as can increasing cementum apposition at the tooth root, which increases with age [54].

In 2020, Rachwal et al. conducted a pilot study to determine whether buccal bone thickness influences the evaluation of root canal visibility in age assessment [55]. The authors acknowledged that their small sample (*n* = 17) was not sufficient to determine a dependence of age on buccal bone thickness in the area of the third molars. The authors concluded by stating that positioning errors in the DPR may well have led to site differences in the feature, as achieving perfect symmetry in the DPR is very difficult. Rachwal et al. saw potential advantages in using cone beam computed tomography (CBCT) [55]. However, age has no influence on the bone thickness of the whole bone in the jaw angle [56]. This is important for using DPR, because, as a summation image, lingual and buccal bone on both sides of the root canal probably determine its visibility. Overall, decreases in radiographic visibility of third molar root canals thus seem to summarize a multitude of age-associated degenerative dental changes and furthermore probably remain strongly dependent on positioning errors of the examined person in the DPR.

Two different stage classifications are available in the literature for evaluating the visibility of the third molar root canals in the DPR. In 2010, a first staging was presented by Olze et al. In 2017, Lucas et al. presented their own new classification where they themselves called it a modification of the Olze method [40, 50]. A group of authors around Al Qattan, which included Lucas herself, published a slightly modified version of the method in 2020, in which a specified inaccuracy of the 2017 version was eliminated [49]. This revised version from 2020, referred to in this report and Al Qattan et al. themselves, as the “Lucas method,” is the basis for the present work.

How the two methods of Olze and Lucas compare and whether one classification is superior to the other need to be thoroughly investigated. The present study, therefore, compared the two classifications in terms of the correlation of stages with age and the reliability of each classification.

## Material and method

The DPRs came from a university dental clinic (Münster, North Rhine-Westphalia region, Germany). The selection of the sample for the present comparison study was guided by pertinent studies within the extant literature of the subject domain. Subsequently, the current investigation aimed to achieve a cohort size of 200 cases, taking into account all age cohorts [23, 57]. In order to accommodate for potential subsequent exclusions, the targeted number of DPRs was initially increased to a total of 300. Inclusion criteria were that the quality of the images had to support assessment of the roots of teeth 38 [FDI] and 48. In addition, the teeth had to be completely mineralized, as determined by attaining stage H of the tooth development scale by Demirjian et al. [22]. Moreover, the teeth had to be free of pathologies such as caries or restorations.

The dental clinic comprised different departments. Therefore, the study population was composed of patients from dental surgery, orthodontics, prosthodontic, and conservative dentistry. In addition, the images, which had all been captured for a medical indication, were retrospectively evaluated in terms of the present research question.

The age of the participants at the time of the X-ray examination had to be known beyond doubt. Radiographs were assessed in DICOM format using synedra Personal View software version 22.0.0.1 (synedra information technologies GmbH, Innsbruck, Austria) at appropriate radiology workstations. The technical setup as well as the ambience was the same for all three examiners. For the evaluations, the software’s magnification tool and the gray-level adjustment tool were routinely used. The examiners were three board-certified dentists.

The evaluations were performed according to the following classifications:

- Olze et al. (2010) (“Olze”) [40]
  - Stage 0 = the lumen of all root canals is visible all the way to apex
  - Stage 1 = the lumen of one root canal is not fully visible to the apex
  - Stage 2 = the lumen of two root canals are not fully visible to the apex, or one canal may be virtually invisible in full length
  - Stage 3 = the lumen of two root canals is virtually invisible in full length

- Al Qattan et al. (2020) (“Lucas”) [49]
  - RPV-A: > 75% of root pulp visible
  - RPV-B: 75 to 50% of root pulp visible
  - RPV-C: 50 to 25% of root pulp visible
  - RPV-D: < 25% of root pulp visible
- [RPV = root pulp visibility]

Prior to the study, 50 independent images were randomly selected for training by the three examiners. Cases with a difference between examiners of more than one stage were discussed together and a common stage was determined. These evaluations were considered the benchmark for the actual determinations.

In the study, radiographs were evaluated in random order and independently. One examiner evaluated the entire dataset a second time. Data management and statistical analyses were performed in Stata, version 13.0 (Stata Corp LP, College Station, TX, USA). Tooth 38 and 48 staging was investigated as means of potentially classifying persons of unknown age into age groups. The distribution of ages was subsequently compared across the stages of each method. Spearman’s rank correlation coefficient evaluated the correlation between age and stage. The degree to which rating might explain the variation in age for each sex was assessed through the adjusted coefficient of determination (adj-*R*^2^) from the regression of age on stage for each method and sex, with adjustment for tooth, and the specific proportion of variance explained (*ω*^2^) by rating. The repeatability of one rater examining each image twice in a random order and the reproducibility of all three raters rating each image once were investigated for each method as means of evaluating the reliability of each method. Fleiss’ kappa, *k*, and Krippendorff’s alpha [58], *α*, were evaluated as measures of agreement between (reproducibility) and within (repeatability) raters.

## Results

According to the inclusion criteria, 233 DPRs from 116 females and 117 males aged 20.0 to 40.9 years were deemed acceptable for assessment in the study. Table 1 shows the composition of the study population according to age and sex.

**Table 1:** Age and sex distribution of the cases included

Age	Female (*n*)	Male (*n*)
20	4	7
21	8	6
22	6	8
23	11	9
24	5	8
25	13	4
26	5	6
27	4	5
28	4	4
29	4	4
30	4	5
31	5	5
32	5	5
33	5	5
34	4	5
35	4	5
36	5	5
37	5	5
38	5	6
39	5	5
40	5	5
Total (*n*)	116	117

For both classifications, all stages were detected in the studied cohort.

Both methods demonstrated only a weak correlation between stage and age (Table 2). Using the Olze method, the Spearman coefficient was higher for males (*ρ* = 0.388 [95% CI 0.309, 0.462]) than for females (*ρ* = 0.283 [95% CI 0.216, 0.357]). The correlation between age and stage was lower in the Lucas method, showing as well a lower correlation for males (*ρ* = 0.212 [95% CI 0.141, 0.284]) than for females (*ρ* = 0.265 [95% CI 0.193, 0.340]). According to the *ω*^2^ statistics from the regression of age on stage, the variation in age between the tooth data could be better explained by the Olze method in males (0.155 [95% CI 0.102, 0.202]) and females (0.141 [0.092, 0.189]), than by the Lucas method (0.045 (0.015, 0.078) and 0.092 (0.050, 0.134) for males and females, respectively.

**Table 2:** Spearman’s correlations between stage and age for each method and sex with the adjusted *R*^2^ coefficient and partial omega-squared (*ω*^2^) value for the stage from the regression of age on stage for each method and sex, adjusted for tooth (95% confidence intervals)

Sex	Method	Spearman’s *ρ* (95% CI)	Adjusted *R*^*2*^	Partial *ω*^2^ (95% CI) for stage, adjusted for tooth
Male	Olze	0.388 (0.309, 0.462)	0.153	0.155 (0.102, 0.202)
	Lucas	0.212 (0.141, 0.284)	0.044	0.045 (0.015, 0.078)
Female	Olze	0.283 (0.216, 0.357)	0.140	0.141 (0.092, 0.189)
	Lucas	0.265 (0.193, 0.340)	0.091	0.092 (0.050, 0.134)

The coefficient for intra-rater repeatability by our single examiner was higher for the Olze method than the Lucas method in the assessment of male third molars with Krippendorff *α*’s of 0.576 [95% CI 0.508, 0.644] and 0.442 [95% CI 0.382, 0.502], respectively (Table 3). Overall repeatability was higher for the Olze method, although in assessing female third molars, repeatability using the Olze method was only slightly greater (*α* = 0.592 [95% CI 0.523, 0.661]) than when using the Lucas method (*α* = 0.516 [95% CI 0.454, 0.578]).

**Table 3:** Fleiss’ kappa, *k*, and Krippendorff’s alpha, *α*, (95% confidence intervals) coefficients measuring intra-rater repeatability for each method applied to each sex

Sex	Method	*κ* (95% CI)	*α* (95% CI)
Male	Olze	0.582 (0.511, 0.652)	0.576 (0.508, 0.644)
	Lucas	0.436 (0.372, 0.500)	0.442 (0.382, 0.502)
Female	Olze	0.586 (0.513, 0.659)	0.592 (0.523, 0.661)
	Lucas	0.511 (0.444, 0.578)	0.516 (0.454, 0.578)

The coefficient measuring inter-rater evaluation of all three examiners was higher for evaluations using the Olze method for both sexes compared to the Lucas method (Table 4). Evaluating the male third molars, the value for Krippendorf’s *α* of 0.542 (95% CI 0.463, 0.620) from the Olze method was considerably higher than that from the Lucas method at 0.374 (95% CI 0.304, 0.443). Among females, this difference between methods was smaller with a Krippendorf *α* of 0.533 (95% CI 0.451, 0.615) for Olze method compared to 0.432 (95% CI 0.359, 0.505) for the Lucas method.

**Table 4:** Fleiss’ kappa, *k*, and Krippendorff’s alpha, *α*, (95% confidence intervals) coefficients measuring inter-rater repeatability for each method applied to each sex

Sex	Method	*κ* (95% CI)	*α* (95% CI)
Male	Olze	0.562 (0.485, 0.640)	0.542 (0.463, 0.620)
	Lucas	0.384 (0.313, 0.454)	0.374 (0.304, 0.443)
Female	Olze	0.544 (0.462, 0.626)	0.533 (0.451, 0.615)
	Lucas	0.445 (0.372, 0.518)	0.432 (0.359, 0.505)

## Discussion

While there was only low correlation of both methods with age, our study found that age-related differences between the third molars could be more reliably evaluated with the Olze method than the Lucas method for both sexes, although this superiority was diminished when evaluating the third molars from females. The present study was explicitly intended to be a comparative study of the two methods under evaluation; it was not intended to provide a reference study for the characteristic of root canal visibility in the DPR. Therefore, statistical measures for the individual stages were omitted. To the best of our knowledge, this study is the first direct comparative study of the two methods.

**Fig. 1:**
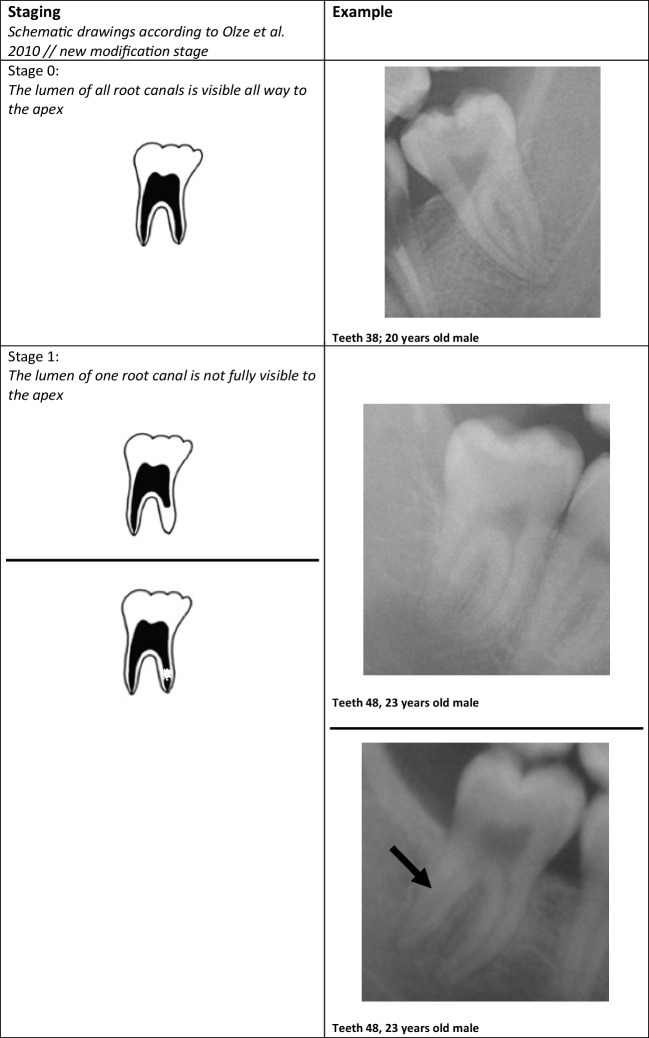

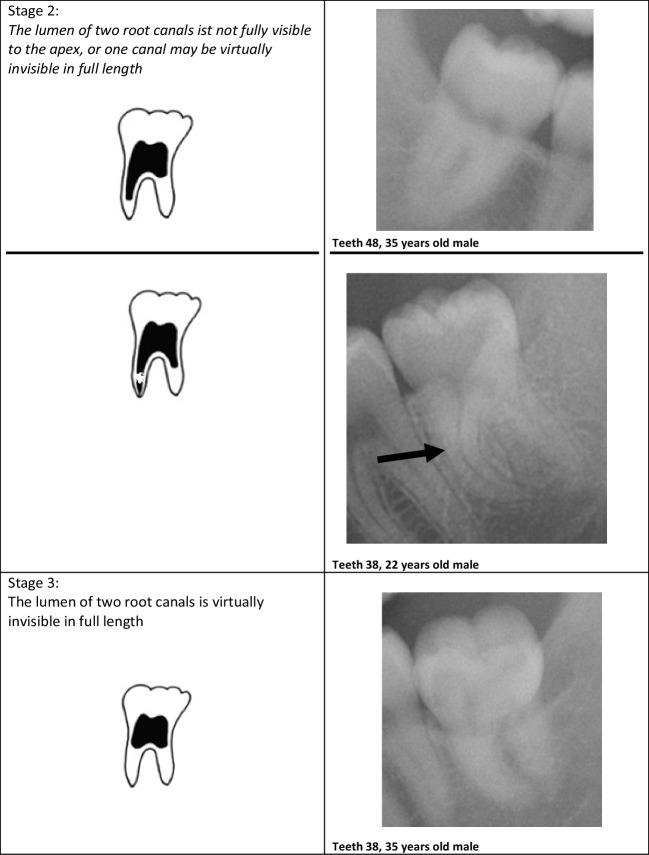
Staging according to Olze et. al (2010) with new additional modifications. Examples from the present cohort. The black arrows show invisible areas in the course of the root

The method of Lucas et al. from 2017 was used as modified by Al Qattan et al. from 2020 [49]. In the original 2017 version, stage RPV-A was defined as “100% of root pulp visible” and stage RPV-D as “0% of root pulp visible” [50]. Stages RPV-B and RPV-C corresponded to the stages as applied in the present study. It was clear that in the 2017 version, the percentages from 0 to 25% and from 75 to 100% did not occur. Thus, the 2017 version is not practicable. Only the version published by Al Qattan et al. overcomes this constraint, which is why this method was also considered in the present study. Besides, both Lucas et al. and Al Qattan et al. used the original pictograms of Olze et al. to visualize the stages [40, 49, 50]. In the 2010 definition, Olze et al. stated “not fully visible” as a criterion in the written definition of the stages [40]. The corresponding pictogram showed a root canal that was not fully visible in the apical region. However, we found cases in which one or both root canals were not fully visible, whereby the invisible area was within the canal course and not only apically. Following the written definition of the stages, these cases were also evaluated as not fully visible for the corresponding stage. This was done especially in view of the fact that the phenomenon’s cause has not been clarified, and thus, a development from apical to coronal does not necessarily have to be given. Therefore, the pictograms were modified (see Fig. 1). The new pictograms also take into account that the boundary from visible to non-visible is usually not as smooth as in the original pictograms.

The reason for the difference in correlation with age between the two methods is not so easy to determine: the classifications have the same number of stages, and the lowest and highest stages are relatively similar [40, 49]. It can be assumed that the selected percentage steps, which are ultimately oriented to the number of stages with the steps in the 25% range, are not so well suited to represent the characteristic being investigated. Furthermore, the authors’ impression suggests that the final step at the highest stages for both methods has a noteworthy impact. In Lucas’ method, approximately a quarter of the root may still remain visible, while in Olze’s version, the root may be completely invisible.

In the case of reliability, the reason for the observed differences is easier to identify: it is difficult to determine the exact percentages, as required by Lucas. A distinction between 49% and 51% visibility, as would be strictly necessary for Lucas’ method, cannot be made in practice. The approach of Olze et al. is much more practicable. In particular, the approach which makes differentiated statements about the various root canals is more clearly applicable in practice. With Lucas, a percentage value must be given for both root canals combined. This makes a practical application even more difficult in our opinion.

Overall, it must be noted that the values for reliability in the current study were considerably lower than the values from the literature. Although it must be taken into account that in the present study Krippendorff’s alpha was chosen as a rather conservative statistic, the differences are clear [58, 59]. Thus, Lucas et al. in 2017 gave a kappa value of 0.89 for the intra-rater repeatability and 0.95 for the inter-rater reproducibility [50]. Al Qattan in 2020 reported kappa values of 0.86 for their estimated intra-rater repeatability and 0.88 for inter-rater reproducibility of the Lucas method [49]. Olze et al. did not make any statement on the reliability of their method in their 2010 publication [40].

In 2019, Akkaya et al. examined reliability for the Olze et al. method [60]. They found kappa values of 0.817 and 0.67 for the intra- and inter-rater agreement, respectively [60]. In 2017, Timme et al. published a reference study of the Olze et al. method [46]. At that time, they found kappa values of 0.72 (tooth 38) and 0.86 (tooth 48) for intra-rater agreement and a value of 0.67 (both teeth) for inter-rater agreement. The examiners in the 2017 study by Timme et al. were completely different from those in the current study [46]. The data from the 2017 study, in particular the value for the inter-rater agreement, were more similar to the data in the present study than those from Lucas et al. or from Al Qattan et al. [49, 50]. On the other hand, Guo et al. found better agreement statistics for the Olze method in 2018, reporting kappa values of 0.825 and 0.788 for the inter- and intra-rater, respectively [47].

The low values for inter-rater reliability relative to the literature are likely to be due to a combination of factors including differences in sampled populations, imaging setup and quality, experimental conditions, and differences in raters. The most important among these is that we compared three examiners in our study. In the existing literature, typically only two examiners were compared [46, 47, 49, 50, 60]. We assume that our results may, therefore, better reflect the conditions of the real world.

The reason for the lower intra-rater agreement relative to the other published studies remains elusive but is likely to be similarly multifactorial as with inter-rater agreement. Nonetheless, it is noteworthy that, within the referenced works, there exists a singular absence of instances wherein the entire dataset underwent re-evaluation for the explicit purpose of ascertaining intra-rater agreement [46, 47, 49, 50, 60]. In one study, the dataset evaluated for intra-rater agreement was separate and not part of the actual study [49]. Notably, the datasets subject to assessment for intra-rater agreement in the cited studies encompassed a spectrum ranging from 50 to 100 radiographs [46, 47, 49, 60]. In one study, information concerning the second observer and the specific radiographs under scrutiny are absent [50].

Since visibility of root canals is an entirely optical phenomenon, it must be stated that the representation of the root canals ultimately also depends on the imaging method used [61]. The characteristic of visibility of the root canals of the third molars was investigated for the present study in the DPR. The DPR represents a very standardized radiographic procedure [62]. On the other hand, it is reported that the representation of the root canals in the cone-beam-CT (CBCT) is strongly dependent on the scan parameters [61]. Root canals that are invisible with one setting can be detectable after changing the parameters [61, 63]. This fact must therefore be taken into account, since the feature of root canal visibility has also been studied recently in CBCT [64, 65]. In the authors’ view, therefore, the reference values of 2D and 3D methods are not directly transferable. Instead, 3D methods can be used to directly determine the volume of the pulp chamber of a tooth and evaluate it for age assessment [12, 66–71].

## Conclusion

In this comparative study, the 2010 method of Olze et al. showed higher correlation with age and was found to perform marginally better than the 2017 method of Lucas et al. in terms of intra- and inter-rater agreement. Also in light of the fact that the majority of previous studies used the Olze method, preference may be given to the Olze method for conducting further studies.

## Data Availability

The datasets generated during the current study are available from the corresponding author on reasonable request.
